# Integrative Whole-Genome and Epigenome Profiling of cfDNA in Familial Prostate Cancer: Insights from a Pilot Study

**DOI:** 10.3390/biomedicines14040818

**Published:** 2026-04-03

**Authors:** Anna Truda, Angela Cordella, Ilenia De Leo, Armando Di Palo, Roberta Iorio, Simona Marino, Roberto La Rocca, Claudia Collà Ruvolo, Nicoletta Potenza, Maria Ravo, Giovanna Marchese

**Affiliations:** 1 Genomix4Life Srl, 84081 Baronissi, Italy; anna.truda@genomix4life.com (A.T.); angela.cordella@genomix4life.com (A.C.); ilenia.deleo@genomix4life.com (I.D.L.); armando.dipalo@genomix4life.com (A.D.P.); roberta.iorio@genomix4life.com (R.I.); simona.marino@genomix4life.com (S.M.); maria.ravo@genomix4life.com (M.R.); 2 Department of Environmental, Biological and Pharmaceutical Sciences and Technologies, University of Campania “Luigi Vanvitelli”, 81100 Caserta, Italy; nicoletta.potenza@unicampania.it; 3 Genome Research Center for Health-CRGS, 84081 Baronissi, Italy; 4 Department of Neurosciences, Reproductive Sciences and Odontostomatology, University of Naples Federico II, 80138 Naples, Italy; roberto.larocca@unina.it (R.L.R.); claudia.collaruvolo@unina.it (C.C.R.)

**Keywords:** cell-free DNA, allele-specific methylation, biomarkers

## Abstract

**Background:** Familial prostate cancer (PCa) accounts for nearly 20% of all PCa cases and is associated with increased genetic susceptibility and earlier disease onset. However, early detection and risk stratification in genetically predisposed individuals remain challenging. Circulating cell-free DNA (cfDNA) provides a minimally invasive source of tumor-derived genomic and epigenomic information. Integrating multi-omic cfDNA analyses may enhance the discovery of biomarkers relevant to familial PCa biology. **Methods:** We conducted a pilot feasibility study employing whole-genome, strand-specific sequencing of cfDNA from eight patients with familial PCa. A unified analytical pipeline was used to jointly profile genomic alterations and epigenomic features. Variant calling, methylation mapping, and allele-specific methylation (ASM) analysis were performed to identify somatic mutations, characterize epigenetic dysregulation, and explore potential interactions between genetic and epigenetic mechanisms. **Results:** Sequencing revealed 18,878 genetic variants, including 2276 potentially pathogenic alterations. We identified 26 recurrent high-impact mutations, such as stop-gain and start-loss variants, in genes including *MUC4*, *MCM9*, and *SKA3*. Epigenomic profiling demonstrated widespread gene-specific hypermethylation, consistent with transcriptional repression in these loci. ASM events were detected in *TTC22*, *TEX51*, *WDR89*, *LAIR2*, and *SKA3*, suggesting coordinated interactions between somatic variation and epigenetic regulation in familial PCa. **Conclusions:** This proof-of-concept study highlights the feasibility and potential of integrating whole-genome and epigenome profiling of cfDNA to decode the molecular architecture of familial prostate cancer. By jointly capturing genomic alterations and epigenetic signatures, including allele-specific methylation, this multi-omic liquid biopsy approach supports a high-resolution exploration of biologically relevant molecular features. Moreover, this integrated profiling strategy provides a minimally invasive and clinically scalable tool that may substantially improve risk assessment. These findings offer a promising foundation for future validation studies in larger cohorts, with the aim of advancing multi-omic cfDNA analysis as a next-generation technology in the field of precision oncologic epigenetics.

## 1. Introduction

Prostate cancer is one of the most common malignancies among the male population worldwide. Nowadays, prostate cancer detection is aided by the measurement of Prostate-Specific Antigen (PSA). Its use as a serum marker has revolutionized prostate cancer diagnosis [1,2]. However, PSA is organ-specific but not cancer-specific; in fact, its levels can also be elevated in benign prostatic hyperplasia (BPH), prostatitis, and other non-malignant conditions. As a result, PSA testing alone is not sufficient to diagnose clinically significant prostate cancer, especially in patients with a family history of the disease. In fact, a man with familial risk may develop aggressive tumors even at relatively low PSA values, leading to underdiagnosis or delayed detection. Therefore, the identification of molecular biomarkers, such as circulating tumor DNA (ctDNA) or cell-free DNA (cfDNA) alterations, could provide a more precise and individualized assessment of cancer risk and disease aggressiveness in this high-risk population [3,4]. Prostate cancer presents as a heterogeneous disease with highly variable clinical outcomes. While men diagnosed with early-stage prostate cancer have a 99% chance of survival, those identified at an advanced stage face a much lower 5-year survival rate of just 31% coupled with a noticeable deterioration in their quality of life [5,6,7]. These pronounced disparities in survival rates underscore the urgent necessity for biomarkers capable of effectively identifying prostate cancer at an early stage and reliably distinguishing its level of aggressiveness [8,9]. Prostate cancer is primarily driven by genetic alterations. These changes in normal prostate cells can lead to disruptions in gene regulation or result in the loss of gene function. Indeed, several gene mutations have been shown to correlate with PCa onset and evolution, offering fundamental information about tumor aggressiveness and therapy response [10,11,12]. One of the most relevant forms from a genetic perspective is familial prostate cancer, which accounts for approximately 20% of all diagnosed cases and is characterized by the recurrence of disease within the same family. Familial prostate cancer is characterized by an early onset age and a more aggressive progression and presents at a more advanced local stage compared to sporadic cases. Additionally, men with familial prostate cancer face a greater likelihood of recurrence following surgery. However, overall survival rates are generally similar between familial and sporadic prostate cancer [13,14]. Although pathogenic germline mutations—primarily in the *BRCA1*, *BRCA2*, *HOXB13*, *ATM*, and *CHEK2* genes—have been identified in 5–10% of patients [15,16,17,18,19,20,21], the overall genetic contribution remains largely unexplained. Having a first-degree relative affected by prostate cancer increases an individual’s relative risk by approximately 2–3 times, and this risk increases further in the presence of multiple affected relatives or early-onset diagnosis [22,23]. While germline alterations underpin hereditary predisposition, somatic mutations acquired during life, as well as epigenetic mechanisms, play a critical role in disease progression, contributing to genomic instability, the emergence of aggressive tumor clones, and therapeutic resistance. Traditionally, tissue biopsies provide important molecular information for diagnosis and prognosis. However, due to their invasive nature, limited ability to capture full tumor heterogeneity, and poor applicability for longitudinal monitoring, they are suboptimal, particularly in diseases where early and dynamic surveillance is essential. In this context, liquid biopsy—and specifically the analysis of circulating cell-free DNA (cfDNA)—has emerged as a minimally invasive approach to access tumor molecular information through a simple blood draw. cfDNA is a fraction of circulating nucleic acid that exists mainly as double-stranded DNA in body fluids [24,25]. It can enter the bloodstream through various processes, including cell lysis, programmed cell death (apoptosis), necrosis or active secretion of DNA fragments into circulation [26,27,28,29]. A fraction of cfDNA may derive from tumor cells and is commonly referred to as circulating tumor DNA (ctDNA); however, ctDNA is intermixed with cfDNA originating from non-tumor cells and cannot be physically isolated with absolute specificity. The proportion of ctDNA in cfDNA varies widely, ranging from less than 0.1% to more than 90% [30,31]. Currently, the tumor-associated features of ctDNA, including point mutation, copy number variations (CNVs), and specific methylation patterns, are of great importance to determine its presence and clinical application [32,33]. In fact, several studies have shown that cfDNA analyses have largely focused on the identification of somatic variants, but recent research has highlighted the added value of integrating epigenetic signatures, particularly cytosine modifications such as 5-methylcytosine (5mC) and 5-hydroxymethylcytosine (5hmC). The innovation of this work consists of the application of new methodologies that allow the simultaneous analysis of genetic and epigenetic components at single-nucleotide resolution. This technology enables the investigation of complex phenomena such as Variant-Associated Methylation (VAM), which refers to the direct association between a genetic variant and an alteration in the DNA methylation profile, either in proximity to the variant or at distant genomic loci. The integrated approach allows for a direct correlation between mutation and methylation, enabling the study of dynamic interactions between genotype and epigenotype [34,35].

In particular, 5hmC is emerging as a dynamic epigenetic biomarker involved in the early stages of tumorigenesis, including prostate cancer also. Additionally, Allele-Specific Methylation (ASM)—defined as the differential methylation between the two alleles of a gene—represents a further layer of epigenetic regulation that is critical to understanding tumor biology [36,37,38]. Genetic variants can influence transcriptional regulation through methylation-dependent mechanisms, a phenomenon increasingly recognized in cancer biology. Recent studies have demonstrated that DNA methylation can modulate the regulatory effects of genetic variants on gene expression, thereby shaping disease-associated transcriptional programs [39]. This study aims to assess the feasibility and potential of an innovative approach based on whole-genome sequencing for the analysis of circulating free DNA (cfDNA). Furthermore, previous evidence has demonstrated allele-specific regulatory activity at prostate cancer risk loci [40], thereby supporting the biological plausibility of the variant-associated methylation effects observed in the present study.

**Figure 1:**
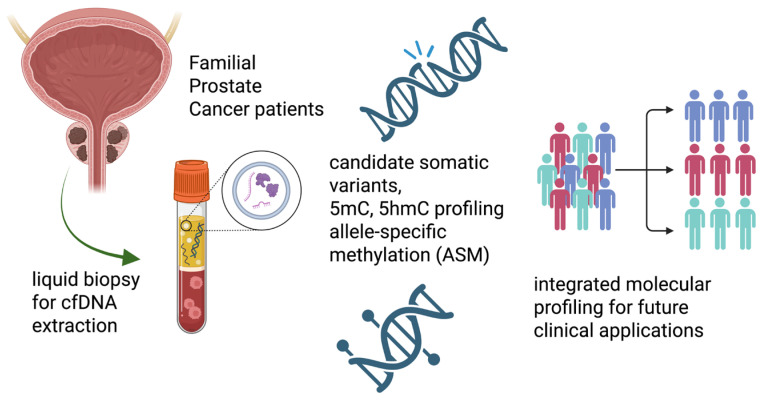
Multi-omic liquid biopsy workflow in familial prostate cancer (pilot study). cfDNA isolated from plasma enables integrated genomic and epigenomic analysis, including candidate somatic variants, 5mC, 5hmC, and allele-specific methylation, providing an exploratory, hypothesis-generating framework for the identification of candidate molecular features.

The strategy integrates the detection of somatic variants with comprehensive epigenetic profiling, including markers such as 5-methylcytosine (5mC), 5-hydroxymethylcytosine (5hmC), variant-associated methylation, and allele-specific methylation. To assess both the feasibility and the informational value of this approach, a pilot study was conducted in patients with familial prostate cancer. The combined analysis of putative tumor-associated variants and high-resolution epigenetic profiles enables the identification of potential molecular signatures characteristic of the disease. The simultaneous detection of genetic and epigenetic alterations from a single minimally invasive cfDNA sample demonstrates the potential clinical applicability of the method and provides a comprehensive view of the molecular landscape of familial prostate cancer. A primary objective of the study is to illustrate the feasibility and potential utility of a multi-omic liquid biopsy approach, highlighting its innovative nature and suggesting possible future applications within the field of precision oncology. To our knowledge, no studies have yet explored the simultaneous analysis of genetic and epigenetic information (5mC and 5hmC) from the same cfDNA sample for biomarker discovery. This approach, combining whole-genome sequencing with integrated analysis of candidate somatic variants and complete epigenetic profiles, thus represents a novel approach for exploring candidate molecular features in familial prostate cancer. Furthermore, we aim to evaluate whether this multi-omic strategy can support the identification of candidate genetic and epigenetic features associated with the disease. In this context, the approach provides a framework for future studies to define molecular profiles and explore disease dynamics. Overall, this work provides a proof of feasibility for the combined analysis of genomic and epigenomic features in cfDNA, identifying candidate regions with coordinated molecular signals. While no clinical conclusions can be drawn at this stage, these results may inform the design of future studies aimed at evaluating potential clinical relevance. (Figure 1).

## 2. Materials and Methods

### 2.1. Patient Cohort and Clinical Data Collection

A total of eight patients with a confirmed diagnosis of prostate cancer were enrolled in this pilot study. All individuals reported a positive family history of cancer, predominantly involving first-degree relatives such as fathers and brothers with prostate cancer. One patient also reported an affected uncle (Supplementary Table S1). The average age at diagnosis was 67.9 years (range: 54–77 years). Pre-treatment serum prostate-specific antigen (PSA) levels ranged from 4.0 to 14.1 ng/mL, with a mean value of 9.9 ng/mL. Prostate volume ranged from 18 to 46 cc (mean: ~36 cc). All patients presented with a clinical stage of T1c or T2a. Magnetic resonance imaging assessment revealed a PIRADS score ≥ 4 in 7 out of 8 patients (87.5%), while one patient had a PIRADS score of 3. The most frequent final pathological stage after radical prostatectomy surgery was pT2 NX MX. A histological cribriform growth pattern was identified in 3 patients (37.5%), all of whom had PIRADS ≥ 4 lesions. Lymphovascular invasion was documented in 4 cases, 3 of which were associated with PSA ≥ 10 ng/mL, and 2 also exhibited cribriform morphology. Extracapsular extension was observed in 2 patients, while seminal vesicle invasion was noted in one patient (PC24). Overall, the clinical data are consistent with features typically observed in prostate cancer, including elevated PSA levels, high PIRADS scores, lymphovascular invasion, and cribriform architecture. At this stage, the patients included in this pilot study have not yet been screened for pathogenic germline mutations (e.g., *BRCA1/2*). All blood samples were collected on the day of surgery before the procedure began. Informed consent was obtained from all subjects at the time of their hospital admission prior to participation in the study. The study was approved by the “Federico II” University Ethics Committee (Comitato Etico Campania 3, Prot. N. Cam3 (Ex DM 8/2/2013)-520/24).

### 2.2. Patients’ Blood Collection and Cell-Free DNA Extraction

Peripheral whole blood samples were collected from eight patients with familial prostate cancer using Cell-Free DNA BCT tubes (Streck, La Vista, NJ, USA) according to the manufacturer’s protocol. Plasma was separated by double centrifugation, first at low speed to remove cells and subsequently at high speed to eliminate any remaining cellular debris, thus obtaining cell-free plasma and minimizing cfDNA degradation. Plasma was then aliquoted into sterile tubes and stored at −80 °C until cfDNA extraction. Cell-Free DNA (cfDNA) was extracted using the QIAamp Circulating Nucleic Acid Kit (Qiagen, Hilden, Germany) according to the manufacturer’s protocol. For cfDNA quantifications, the Cell-free DNA ScreenTape assay (Agilent Technologies, Santa Clara, CA, USA) and Qubit DNA HS assay kit (Thermo Fisher Scientific, Waltham, MA, USA) were used. Tumor fraction was not directly estimated in this study, as cfDNA was extracted and analyzed as a whole without physical separation of circulating tumor DNA (ctDNA) from cfDNA.

### 2.3. cfDNA Sequencing

Indexed libraries were prepared from 10 ng purified total cfDNA each using Biomodal duet evoC kit (Biomodal, Chesterford Research Park, Cambridge, United Kingdom) according to the manufacturer’s instructions. Duet Multiomics + modC solution is a bimodal approach that simultaneously captures both genetic and epigenetic information (5mC and 5hmC) from the same DNA molecule in a single workflow. In brief, fragmented genomic DNA is ligated to hairpin adapters, forming a hairpin complex that is split into two strands. A complementary copy strand is synthesized to preserve the original genetic sequence. Modified cytosines are protected via oxidation, while unmodified cytosines are deaminated to uracil. After ligating sequencing adapters, the hairpin is linearized, PCR amplified, and sequenced. Read 1 sequences the original strand, and Read 2 sequences the complementary strand. A bespoke biomodal pipeline aligns complementary reads and resolves each base, simultaneously determining genetic variants and cytosine modifications. Sequencing or PCR errors are filtered out. This approach preserves all four canonical bases for alignment while capturing both 5mC and 5hmC in a single assay, allowing integrated, phased analysis of genetic variants, DNA methylation, 5hmC profiles, and variant-associated methylation from limited DNA input such as cfDNA, without the limitations associated with traditional bisulfite-based methods. For library quantifications, the Tape D5000 assay kit (Agilent Technologies, Santa Clara, CA, USA) and Qubit DNA HS assay kit (Thermo Fisher Scientific, Waltham, MA, USA) were used. Indexed libraries were pooled in equimolar amounts with a final concentration of 1,5 nM. Illumina NovaSeq 6000 System was used to sequence the pooled samples in a 2 × 151 paired-end format on an S4 300-cycle flowcell (Illumina, San Diego, CA, USA). Regarding sequencing depth, according to the protocol, 8 samples were loaded on an S4 flowcell, resulting in a total output of 20 billion reads. For each sample, approximately 2.5 billion reads were generated, achieving an average coverage of around 120X.

### 2.4. Data Analysis

Raw sequencing data were processed using Nextflow Duet pipeline version 1.3.0, which integrates genetic and epigenetic information by aligning and merging original and complementary DNA strand sequences. Cutadapt v4.7 was used to remove hairpin structures and sequencing adapter sequences, followed by quality filtering to generate high-quality FASTQ files [41]. Reads were aligned to the reference genome (GRCh38Decoy with Gencode v40 annotation) and spike-in controls using BWA-MEM v2.2.1 [42]; duplicates were removed with Picard MarkDuplicates v4.5.0.0 (about 30% for all samples). Methylation status at each CpG site was quantified, with accuracy assessed using spike-in controls. Somatic variants were called with GATK Mutect2 v4.5.0.0 in tumour-only mode. Subsequently, to remove false positives and incorrect calls of germline variants, the pipeline included the execution of the ‘FilterMutectCalls’ module (https://gatk.broadinstitute.org/hc/en-us/articles/360036856831-FilterMutectCalls, accessed on 22 February 2024) [43,44,45]. Somatic origin was inferred through multiple layers of statistical evidence, including germline risk assessment (GERMQ and population allele frequencies), filtering against a Panel of Normals (PoN), and likelihood-based scoring (Tumor LOD). Only variants passing all filters were retained in the final VCF files for downstream analyses. Epigenetic calls were preserved from FASTQ to BAM files using MM tags. Outputs included unannotated VCF files, BAM files with MM tags, and duet Cytosine reports for epigenetic quantification. Variant annotation was performed with ANNOVAR v2020.06.0 [46] (version dated 7 June 2020), utilizing databases such as refGene [47] (transcript annotation, 17 August 2020, UCSC), ClinVar [48] (release 20240917), gnomAD [49] (gnomad41_genome allele frequencies), COSMIC [50] and multiple predictive scores from dbNSFP [51]. The Duet pipeline provides quantitative allele-level information for each heterozygous variant or phased haplotype, including per-allele read counts, the number of methylated and unmethylated CpG sites, and allele-specific methylation fractions. For each site, allele-specific methylation (ASM) was assessed using dedicated statistical tests, with *p*-values adjusted for multiple testing to estimate the confidence of ASM calls. Quality and coverage criteria were also applied, retaining only sites supported by at least six reads per allele. Sites showing a methylation difference below 30% were classified as “low difference”, whereas loci displaying distinct allele-specific methylation patterns were categorized as “high”, “low”, or “other” according to the classification provided by the pipeline. The “other” category was used to identify candidate ASM sites with potential biological relevance. Methylation status at each CpG site was quantified, with accuracy assessed using spike-in controls. In particular, the pipelines begin epigenetic analysis once the aligned, lane-merged BAM files have been filtered. By default, the assessment of epigenetic status is carried out at CpG sites, using both genome-aligned reads and control data. Quantification is restricted to CpGs annotated in the reference genome and therefore does not account for CpG sites that are unique to the individual sample but absent from the reference sequence. The sensitivity of mC is calculated as the fraction of total calls relative to the expected mC calls in the lambda genome; the sensitivity of hmC is calculated as the fraction of total calls relative to the expected hmC calls on one of the short oligo controls; specificity is calculated as the fraction of total calls relative to the expected C calls in pUC19; finally, modC sensitivity is calculated as the fraction of calls that are mC, hmC, or undifferentiated modC relative to the expected mC calls in the lambda genome. The data are publicly available at ArrayExpress under accession number E-MTAB-15437.

## 3. Results

### 3.1. Somatic Variants Identified in cfDNA

**Figure 2:**
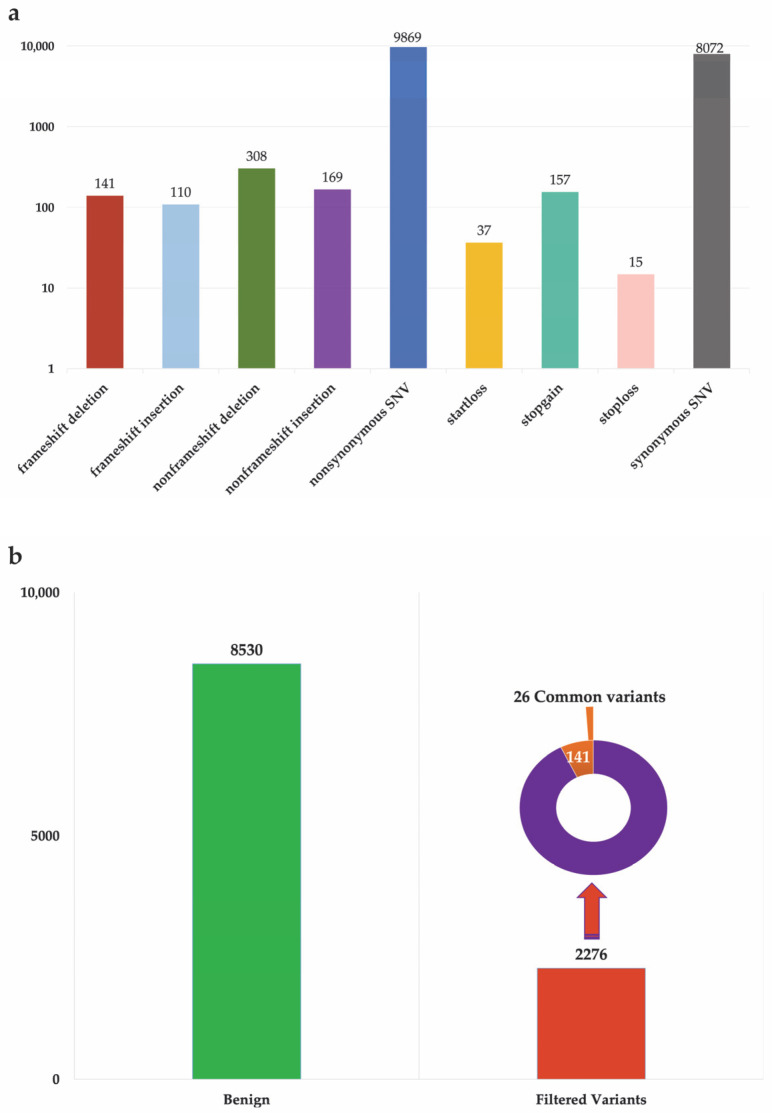
Distribution of somatic variants by type of functional impact. (**a**) The chart shows the number of identified variants categorized by type: frameshift (insertions and deletions), nonframeshift, synonymous and nonsynonymous SNVs (Single Nucleotide Variants), startloss, stopgain, and stoploss. (**b**) Predicted functional impact of variants: benign (*n* = 8530) and filtered variants (*n* = 2276), based on in silico prediction tools. Among these, a subset of high-impact variants stands out: nonsense mutations found in all samples (*n* = 141) and 26 present in at least two samples (*n* = 26). The y-axis is displayed on a logarithmic scale.

A total of 18,878 genetic variants were identified from the analysis using gnomAD v4.1 allele frequency (AF) values < 0.05 (Figure 2a). The vertical bar chart depicting the distribution of these genetic variants reveals a notable predominance of nonsynonymous and synonymous variants, with 9869 and 8072 events, respectively. Nonsynonymous SNVs were the most frequent category; although their precise role remains to be clarified, some of these variants may have a significant impact on protein function and represent an interesting avenue for future studies in the analyzed biological context. Among the indels (insertions and deletions), nonframeshift deletions were the most frequent, with 308 events, followed by nonframeshift insertions (169), frameshift deletions (141), and frameshift insertions (110). Although less numerous, frameshift variants are generally associated with more significant effects on the resulting protein, as they alter the reading frame of the genetic message. Variants directly affecting start and stop codons, although less frequent, are of particular functional interest. A total of 157 stopgain, 37 startloss, and 15 stoploss variants were observed, all potentially associated with premature truncations or, conversely, abnormal elongations of the encoded proteins (Figure 2a). After excluding synonymous variants, 10,806 variants were retained (Figure 2b). Of these, 8530 were classified as benign, while the remaining 2276 were considered potentially pathogenic, being annotated as “damaging,” “probably damaging,” “possibly damaging,” or “disease causing”. The selection of potentially pathogenic variants was performed through the integration of multiple functional prediction tools. Specifically, variants annotated as “Tolerated” in the SIFT and SIFT4G databases, those classified as “Benign” by PolyPhen-2 (both HDIV and HVAR models), and variants labelled as “Polymorphism” or “Non-disease causing” by MutationTaster were excluded. The combined application of these filters resulted in a final set of 2276 variants with potential functional impact (Figure 2b, Filtered Variants).

Among filtered variants, 141 variants were classified as high-impact mutations, specifically start-loss, stop-gain and stoploss variants, while the remaining 2135 included other types of mutations, primarily missense and frameshift variants. Analysis of the distribution of the 141 start-loss/stop-gain variants revealed that 26 of them were shared by at least two patients, suggesting a potentially recurrent or common role in the pathological context under investigation. As shown in Table 1, our analysis focused on these 26 genetic variants, including single-nucleotide polymorphisms (SNPs), insertions and deletions. It is important to note that alterations were identified in many genes of significant functional and clinical relevance, although not in all genes, as some, including an ORF and a LOC, have not yet been functionally characterized. The analysis highlighted mutations in several genes potentially involved in tumorigenic processes or in key biological pathways critical for cell growth and survival.

### 3.2. Epigenetic Profiles and Allele-Specific Methylation

**Figure 3:**
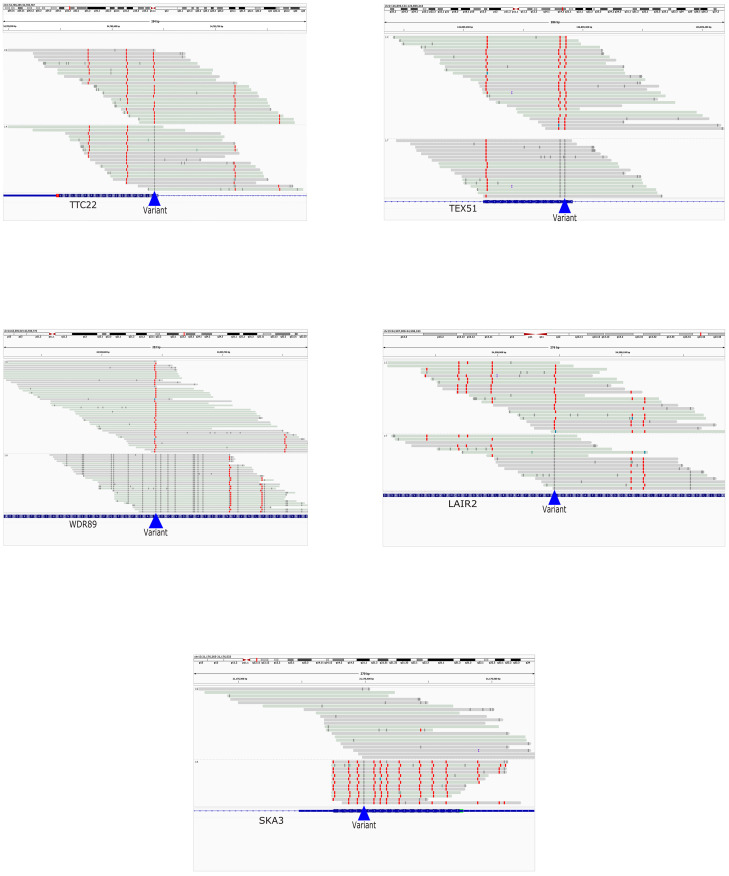
Visualization of somatic variants and allele-specific methylation (ASM).

To complement the genomic findings with epigenomic insight, we examined cytosine modifications, specifically 5-methylcytosine (5mC) and 5-hydroxymethylcytosine (5hmC), within the 26 genes harboring the selected somatic variants. The analysis revealed high levels of DNA methylation, as shown in Table 2, suggesting that epigenetic mechanisms could play a role in the regulation of gene expression in response to somatic alterations. This widespread hypermethylation pattern may indicate transcriptional silencing of key loci involved in cancer biology. Further investigation was conducted to assess whether specific genetic variants were associated with localized changes in DNA methylation, both at the exact variant sites and within the flanking genomic regions. Special attention was given to Allele-Specific Methylation (ASM), defined as differential methylation between the two alleles of a gene (Supplementary Table S2). ASM represents an additional layer of regulatory complexity, potentially influencing gene expression in a variant-dependent manner. Notably, *TTC22*, *TEX51*, *WDR89*, *LAIR2*, and *SKA3* exhibited distinct allele-specific epigenetic profiles, as visualized in Figure 3, suggesting a functional role of ASM at these loci. These genes demonstrated pronounced allelic differences in methylation, reinforcing the hypothesis that genetic variation can modulate epigenetic states, ultimately contributing to tumorigenesis (Figure 3). These findings emphasize the importance of integrating genomic and epigenomic data to better understand the molecular complexity of familial prostate cancer. The raw data supporting these analyses are publicly available at ArrayExpress under accession number E-MTAB-15437.

Somatic variants and allele-specific methylation (ASM) in *TTC22*, *TEX51*, *WDR89*, *LAIR2*, and *SKA3* were visualized using IGV (Integrative Genomics Viewer). Sequencing reads are displayed in grey, with methylated cytosines highlighted in red. The figure illustrates the relationship between somatic mutations and methylation patterns at the nucleotide level, enabling visualization of potential allele-specific epigenetic regulation in these genes. Quantitative analyses revealed variable degrees of allelic methylation imbalance across loci, with higher differences observed in genes such as *SKA3* and *TEX51* (methylation difference up to ~0.87 and ~0.71, respectively) and more moderate effects in *WDR89*, *LAIR2*, and *TTC22* (~0.17–0.38). These patterns are supported by allele-specific read counts and statistically significant associations (corrected *p*-values up to <10^−10^ in selected loci). Detailed quantitative metrics, including allele counts, methylation differences, and statistical significance, are provided in Supplementary Table S2.

## 4. Discussion

In this study, we identified 26 genes harboring recurrent stop-gain mutations in at least two patients, suggesting a possible association with tumor-related processes. Stop-gain mutations introduce premature termination codons, often leading to loss of gene function, thereby contributing to carcinogenic mechanisms through the inactivation of genes critical for cellular homeostasis. Among the mutated genes, *MUC4* and *MCM9* stand out for their functional relevance. *MUC4*, a transmembrane mucin broadly expressed in epithelial tissues and frequently deregulated in tumors, is involved in the regulation of cell proliferation, adhesion, and receptor-mediated signaling such as *ErbB2*. Loss of function potentially induced by stop-gain mutations could alter its structural roles and modulate interactions with the tumor microenvironment, promoting neoplastic progression and immune evasion [52,53,54]. Conversely, *MCM9* is a key player in DNA repair via homologous recombination. Its inactivation, for instance, through stop-gain mutations, has been implicated in compromised genomic integrity, leading to increased mutagenesis and chromosomal instability. Previous studies, including CRISPR/Cas9 experiments, have suggested that loss of *MCM9* may sensitize human cells to PARP inhibitors such as Olaparib, positioning it among major factors involved in homologous recombination and interstrand crosslink repair [55,56,57,58]. In prostate cancer, genomic deletions encompassing *MCM9* (e.g., recurrent deletions at 6q in prostate cancer) have been reported to frequently co-occur with alterations in other DNA damage response genes and correlate with increased sensitivity to PARP-targeted therapies [52,55,59,60,61,62]. Although these studies do not directly investigate *MCM9*, they underscore the importance of homologous recombination-mediated DNA repair in response to PARP inhibitors in prostate cancer. Since *MCM9* participates in this pathway, its inactivation could theoretically influence PARP inhibitor sensitivity [56,59,63,64,65,66,67]. It is important to note that our study did not directly assess the potential link between *MCM9* inactivation and PARP inhibitor sensitivity; therefore, this remains a hypothesis requiring further functional validation in future studies. Other mutated genes, such as *PRAMEF2*, *PSRC1*, *SKA3*, and *ZNF77*, are less characterized but potentially involved in cell division, cell cycle regulation, transcription, and immune response. Stop-gain mutations in these genes may be associated with biological processes relevant to tumor development, although their functional impact remains largely unexplored. From an epigenetic perspective, we observed an overall increase in DNA methylation (5mC) in many of the mutated genes. However, it is important to emphasize that a global increase in methylation levels does not necessarily imply transcriptional repression, as the effect depends on the location of the methylated sites: for example, methylation in promoter regions or the first intron is more frequently associated with gene silencing. In five genes (*TTC22*, *TEX51*, *WDR89*, *LAIR2*, and *SKA3*), we identified an allele-specific methylation (ASM) profile, with marked differences between alleles, suggesting a functional interplay between candidate somatic mutation and the epigenome. These observations are consistent with previous studies demonstrating that genetic variation can influence transcriptional regulation through methylation-dependent mechanisms [39] and that DNA methylation may act as a causal regulatory layer in prostate cancer biology [68].

In some cases, such as *TTC22*, *TEX51*, *WDR89*, and *LAIR2*, a C > T mutation may result in the loss of a CpG site, reducing methylation levels and potentially leading to increased gene expression (Figure 3). These findings support the hypothesis that candidate somatic variants can contribute to epigenetic deregulation in tumors. *TEX51*, typically expressed in germline tissues, was also detected in tumors and may be considered a cancer-testis antigen with potential immunotherapeutic implications [69]. *WDR89* and *LAIR2* have been implicated in oncogenic and immune-related processes, respectively, highlighting their potential relevance as candidate loci. *TTC22*, although not yet studied in prostate cancer, has been implicated in other solid tumors: in pancreatic cancer, it is associated with poor prognosis and immune infiltration; in colon cancer, it promotes metastatic progression by upregulating *WTAP* and *SNAI1*, key regulators of epithelial–mesenchymal transition [70,71]. These findings suggest a potential role for *TTC22* in prostate cancer, warranting further investigation. A particularly relevant case is *SKA3*, where the associated variant correlates with hypermethylation across the entire gene region, delineating an ASM profile not previously described in prostate cancer. Since *SKA3* plays a key role in mitosis and chromosomal stability, monoallelic regulation of expression could promote genomic instability and tumor progression. Although direct data on prostate cancer are limited, analogous observations for *KLK3 (PSA)* indicate that monoallelic or biallelic methylation can associate with distinct tumor phenotypes [72,73], supporting the identification of *SKA3* as a candidate locus that may be associated with epigenetic prognostic features, warranting further investigation.

This study has limitations, as the tumor-only design does not allow us to completely exclude the constitutional origin of all variants. To mitigate this risk, we applied stringent filters on VAF, excluded variants compatible with germline origin, and removed potential sequencing artifacts. However, the presence of rare germline variants or mutations associated with clonal haematopoiesis cannot be entirely excluded. To minimize the potential contribution of clonal hematopoiesis (CHIP), the genes identified in cfDNA were compared with known CHIP driver gene lists reported in large-scale studies [74]. None of the prioritized genes in our dataset overlapped with the main CHIP drivers (e.g., *DNMT3A*, *TET2*, *ASXL1*, and *TP53*), suggesting a reduced likelihood of a hematopoietic origin of the variants, although this possibility cannot be completely excluded. Likewise, the limited sample size reduces statistical power, and the observations should therefore be interpreted as preliminary and exploratory. Our data identify candidate loci (*TTC22*, *TEX51*, *WDR89*, *LAIR2*, and *SKA3*) and suggest that integrating genetic and epigenetic profiles from cfDNA may provide an exploratory framework for the characterization of candidate molecular features. However, it is clear that the variants identified in our study should be considered as candidate tumor-associated loci rather than definitive disease-driving alterations, and their potential biological significance needs to be confirmed in larger cohorts and through dedicated functional assays [75,76]. Despite the limitations of this pilot study, the multi-omic liquid biopsy strategy presented here represents a methodologically promising approach, with potential perspectives for clinical application, and may contribute to the advancement of precision medicine.

## 5. Conclusions

This pilot study demonstrates the methodological feasibility of integrating genomic and epigenomic analyses of cfDNA in familial prostate cancer. The multi-omic approach revealed the presence of mutations and altered epigenetic profiles, including allele-specific methylation events, suggesting a possible interplay between somatic variation and the epigenome, the biological relevance of which requires further investigation. Although previous studies, such as Ci et al. [77], have identified clinically relevant methylation signatures in larger and well-characterized cohorts—highlighting the level of validation required for potential clinical application—our study is limited by the small sample size and should therefore be interpreted as an exploratory, proof-of-principle analysis. Overall, our findings provide preliminary indications of the potential of multi-omic liquid biopsy approaches in the study of genetically predisposed tumors. However, these results should be considered preliminary and require validation in larger, independent, and longitudinal cohorts before any clinical translation can be envisaged.

## Figures and Tables

**Table 1:** Somatic Variants Identified in cfDNA. In the table are indicated: Chr (Chromosome); Start–End (genomic coordinates); Ref (Reference allele, the base(s) found in the reference genome at that position), Alt (Alternate allele(s), the observed variant(s) that differ from the reference; Gene (name of gene); Transcript (annotated transcript from RefSeq); HGVSC (position of the variant on the coding DNA reference sequence according to the nomenclature Human Genome Variation Society) HGVSP (position of the variant on the protein reference sequence according to the nomenclature Human Genome Variation Society); N° of patients (number of samples in which the variant was observed); AF (Allele Frequency); Mean Read Depth (the average number of sequencing reads covering a genomic position, calculated across subjects); (*) indicates a stop codon.

Chr	Start	End	Ref	Alt	Gene.	Transcript	HGVSC	HGVSP	N° Patients	AF	Mean Read Depth
chr1	12,860,036	12,860,036	G	T	*PRAMEF2*	NM_023014	c.G631T	p.E211X	3	0.0466	69
chr1	109,280,835	109,280,835	T	A	*PSRC1*	NM_001005290	c.A745T	p.K249X	2	0.0144	40
chr1	54,785,641	54,785,641	G	A	*TTC22*	NM_017904	c.C1024T	p.R342X	3	0.0639	50
chr2	126,899,285	126,899,285	C	T	*TEX51*	NM_001322244	c.C214T	p.R72X	2	0.0629	60
chr3	195,781,189	195,781,189	G	-	*MUC4*	NM_018406	c.10391delC	p.S3464X	2	0.0176	45
chr5	141,433,208	141,433,208	-	T	*PCDHGA12*	NM_032094	c.2450dupT	p.*821L	2	0.0010	50
chr6	17,605,931	17,605,931	C	T	*FAM8A1*	NM_016255	c.C1015T	p.R339X	5	0.0042	47
chr6	32,584,158	32,584,158	G	T	*HLA-DRB1*	NM_002124	c.C321A	p.Y107X	2	0.0094	31
chr6	118,894,558	118,894,558	G	A	*MCM9*	NM_001378365	c.C1918T	p.Q640X	3	0.0573	30
chr6	154,246,729	154,246,729	C	T	*OPRM1*	NM_001008503	c.C1201T	R401X	2	0.0478	50
chr9	136,253,933	136,253,933	-	CCACCAGGCCCAGGCGCCCGGCTCTCAG	*CCDC187*	NM_001378188	c.5894_5895insCTGAGAGCCGGGCGCCTGGGCCTGGTGG	p.N1966*	2	0.0464	49
chr9	127,714,388	127,714,388	G	C	*PTRH1*	NM_001345979	c.C353G	p.S118X	3	0.0235	48
chr10	1,019,770	1,019,770	C	T	*IDI2*	NM_033261	c.G431A	p.W144X	2	0.0582	40
chr11	5,967,993	5,967,993	G	A	*OR56A5*	NM_001146033	c.C502T	p.R168X	2	0.0608	51
chr12	7,322,485	7,322,485	C	T	*ACSM4*	NM_001080454	c.C1069T	p.Q357X	3	0.0587	35
chr13	21,176,399	21,176,399	G	A	*SKA3*	NM_001166017	c.C79T	p.R27X	3	0.0499	32
chr14	63,599,645	63,599,645	G	A	*WDR89*	NM_001258272	c.C298T	p.R100X	6	0.0024	52
chr15	83,008,518	83,008,518	-	AA	*C15orf40*	NM_001160113	c.395_396insTT	p.L132Ffs*2	3	0.0004	30
chr17	41,439,232	41,439,232	G	A	*KRT38*	NM_006771	c.C703T	p.Q235X	5	0.0642	50
chr17	41,054,930	41,054,930	C	T	*KRTAP2-2*	NM_033032	c.G282A	p.W94X	5	0.0539	20
chr17	28,326,780	28,326,780	-	A	*TMEM97*	NM_014573	c.519dupA	p.*177delinsMKETTTGPG*	3	0.0001	40
chr18	7,456,247	7,456,247	G	A	*LOC112577592*	NM_001364581	c.C205T	p.R69X	2	0.0814	56
chr18	46,946,874	46,946,874	T	C	*KATNAL2*	NM_001367621	c.T2C	p.M27T	2	0.0259	45
chr19	54,508,046	54,508,046	C	T	*LAIR2*	NM_002288	c.C226T	p.R76X	2	0.0323	40
chr19	2,936,537	2,936,537	G	A	*ZNF77*	NM_021217	c.C298T	p.Q100X	2	0.0339	37
chr21	10,462,836	10,462,836	C	T	*BAGE3*	NM_182481	c.C280T	p.R94X	6	0.0489	68

**Table 2:** Percentage of 5-methylcytosine (5mC) and 5-hydroxymethylcytosine (5hmC) within the 26 genes harboring the selected somatic variants.

	*ACSM4*			*BAGE3*			*C15orf40*			*CCDC187*			*FAM8A1*		
	%_5mC	%_5hmC	Total_C	%_5mC	%_5hmC	Total_C	%_5mC	%_5hmC	Total_C	%_5mC	%_5hmC	Total_C	%_5mC	%_5hmC	Total_C
PC10	87.59	2.37	9493	2.71	0.19	76,460	71.47	2.92	14,223	78.43	1.96	72,584	47.36	5.43	6611
PC14	87.27	1.69	9002	1.93	0.19	62,899	74.28	2.14	12,841	78.38	1.44	71,011	51.45	4.05	6202
PC24	87.21	1.2	8470	1.38	0.24	70,005	74.95	2.09	11,855	79.25	1.24	59,073	51.57	3.4	5379
PC26	85.62	1.55	9879	1.81	0.2	80,974	73.04	2.74	14,404	77.96	1.56	79,124	49.28	4.01	6863
PC30	87.07	1.47	8861	1.69	0.19	70,914	73.78	2.08	13,512	79.64	1.29	68,732	47.99	3.36	6216
PC4	87.57	1.92	9770	2.2	0.24	72,565	73.35	2.59	13,953	80.51	1.59	76,500	51.96	4.35	6320
PC5	84.71	2.2	8181	2.6	0.28	62,365	62.65	3.57	13,313	75.92	2.33	62,677	37.17	4.51	7313
PC6	87.13	1.32	10,095	1.51	0.15	71,256	68.6	1.97	14,916	78.41	1.27	72,614	43.54	3.56	7609
	***HLA-DRB***			***IDI2***			***KATNAL2***			***KRT38***			***KRTAP2-2***		
	**%_5mC**	**%_5hmC**	**Total_C**	**%_5mC**	**%_5hmC**	**Total_C**	**%_5mC**	**%_5hmC**	**Total_C**	**%_5mC**	**%_5hmC**	**Total_C**	**%_5mC**	**%_5hmC**	**Total_C**
PC10	55.19	1.27	4553	88.6	2.36	8547	79.37	1.24	70,352	83.01	1.26	3815	84.33	0.62	970
PC14	53.8	1.13	4766	89.58	1.8	7900	81.05	0.86	65,683	81.73	1.08	3700	87.93	0.19	1036
PC24	51.27	0.69	4203	89.39	1.38	7379	81.47	0.94	59,876	84.42	0.73	3274	86.02	0.24	830
PC26	47.24	1.9	6463	89.83	1.99	9015	80.8	1.2	72,925	81.26	0.91	4088	85.71	0.38	1057
PC30	50.87	1.08	3613	88.94	1.82	8090	81.38	1.15	63,990	83.24	1.32	3706	87.47	0.11	870
PC4	55.02	0.88	5234	90.31	2.15	9183	81.32	0.97	71,952	82.58	1.15	4184	90.14	0.3	1004
PC5	48.18	1.98	4541	88.26	3.13	7606	76.6	1.76	61,747	78.78	1.1	3284	82.75	0.48	835
PC6	49.36	1.09	5581	89.44	1.45	9145	78.04	0.88	73,326	81.02	0.98	3994	88.32	0.1	976
	***LAIR2***			***LOC112577592***			***MCM9***			***MUC4***			***OPRM1***		
	**%_5mC**	**%_5hmC**	**Total_C**	**%_5mC**	**%_5hmC**	**Total_C**	**%_5mC**	**%_5hmC**	**Total_C**	**%_5mC**	**%_5hmC**	**Total_C**	**%_5mC**	**%_5hmC**	**Total_C**
PC10	76.41	1.39	7842	87.41	1.31	5330	67.56	4.22	43,041	74.49	3	71,288	81.27	2.5	81,156
PC14	77.19	0.96	7400	85.67	1.13	5324	74.12	2.93	39,991	75.08	2.36	72,034	81.76	1.94	77,657
PC24	77.03	0.94	6490	84.37	0.86	4990	74.87	2.5	36,592	75.83	2	59,504	82.84	1.64	73,257
PC26	75.96	1.1	8337	83.99	1.53	5827	71.93	3.08	45,464	73.98	2.55	77,347	80.59	1.66	87,327
PC30	78.04	0.97	7289	85.81	1.03	5512	74.2	2.73	40,129	76.55	2.34	66,747	81.76	1.95	78,238
PC4	78.78	0.89	8397	86.98	1.77	5877	73.87	3.5	43,635	75.51	2.41	78,859	82.93	1.88	85,909
PC5	76.21	1.42	7133	82.7	1.71	4666	61.06	4.04	42,392	71.65	3.14	62,596	78.88	2.49	72,293
PC6	74.08	0.73	7794	86.11	1.36	5963	66.57	2.37	49,622	76.46	2.23	69,939	81.14	1.66	87,572
	***OR56A5***			***PCDHGA12***			***PRAMEF2***			***PSRC1***			***PTRH1***		
	**%_5mC**	**%_5hmC**	**Total_C**	**%_5mC**	**%_5hmC**	**Total_C**	**%_5mC**	**%_5hmC**	**Total_C**	**%_5mC**	**%_5hmC**	**Total_C**	**%_5mC**	**%_5hmC**	**Total_C**
PC10	81.27	0.79	630	56.7	1.5	59,495	76.24	0.73	4117	35.68	5.81	5162	61.94	2.8	18,707
PC14	79.56	0.32	631	57.12	1.18	59,648	79.7	0.57	2970	43.09	5.18	4574	66.62	2.19	17,048
PC24	77.89	1.19	588	59.84	0.9	52,534	83.45	0.49	2840	43.47	4.13	3752	68.33	1.71	14,416
PC26	77.15	0.47	639	58.57	1.34	65,055	77.31	0.81	2838	43.37	4.11	5474	63.82	2.16	18,485
PC30	84.33	0.65	619	60.44	1.11	58,130	80.26	0.61	2953	40.25	5.01	4288	66.01	2.06	16,320
PC4	78.53	0.75	666	60.67	1.31	63,584	76.91	0.86	3820	40.78	5.71	4943	66.54	2.26	18,491
PC5	72.71	0.54	557	54.62	1.83	53,334	74.85	1.05	2282	28.52	5.13	6298	52.31	2.6	18,200
PC6	78.65	0.46	651	60.6	1.14	62,654	81.32	0.82	3185	34.03	3.17	6060	59.22	1.79	19,900
	***SKA3***			***TEX51***			***TMEM97***			***TTC22***			***WDR89***		
	**%_5mC**	**%_5hmC**	**Total_C**	**%_5mC**	**%_5hmC**	**Total_C**	**%_5mC**	**%_5hmC**	**Total_C**	**%_5mC**	**%_5hmC**	**Total_C**	**%_5mC**	**%_5hmC**	**Total_C**
PC10	67.2	2.01	13,529	86.71	0.72	2627	48.6	1.63	8018	70.44	3.02	15,160	72.32	3.18	23,943
PC14	70.95	1.4	12,984	85.81	0.69	2622	53.3	1.78	7404	72.62	2.05	15,187	79.88	2.42	20,772
PC24	72.84	1.06	11,750	87.73	0.87	2306	56.28	1.63	6311	73.87	1.75	13,234	80.47	2.28	19,054
PC26	69.62	1.35	14,267	82.88	1.13	2915	47.9	2.23	8705	70.73	2.13	17,075	78.47	2.71	23,842
PC30	69.97	1.18	13,169	88.13	0.68	2510	48.96	1.31	8016	72.3	1.87	15,058	79.01	2.4	21,806
PC4	72.78	1.47	15,082	88	0.94	2776	52.59	1.74	8035	73.69	2.42	16,794	80.91	2.94	22,423
PC5	59.18	1.88	13,688	83.34	0.89	2239	38.89	2.09	8579	62.15	3.72	14,124	66.91	3.52	22,826
PC6	68.64	1.02	15,042	85.94	0.53	2631	46.76	1.22	9163	72.53	2.09	15,853	72	2.03	25,607
	***ZNF77***														
	**%_5mC**	**%_5hmC**	**Total_C**												
PC10	64.59	1.6	14,167												
PC14	72.5	1.21	12,024												
PC24	73.06	1.37	11,050												
PC26	69.92	1.46	13,917												
PC30	70.87	1.25	12,588												
PC4	74.47	1.82	12,979												
PC5	59.27	1.88	13,691												
PC6	60.11	1.3	15,953												

## Data Availability

Deidentified data supporting the findings of this study have been made available in June 2026.

## Supplementary Materials

The following supporting information can be downloaded at: https://www.mdpi.com/article/10.3390/biomedicines14040818/s1, Supplementary Table S1: Summary of clinical, tumor, and pathological characteristics of the study cohort, including demographic data, PSA levels, MRI findings, staging, and key histopathological features. Supplementary Table S2: Summary of allele-specific methylation (ASM) results, including genomic position, allele-specific metrics, and statistical significance.

## Abbreviations

The following abbreviations are used in this manuscript:

PCa	Prostate cancer
cfDNA	cell-free DNA
ASM	Allele-specific methylation
PSA	Prostate-Specific Antigen
BPH	Benign prostatic hyperplasia
ctDNA	Circulating tumor DNA
CNV	Copy number variations
5mC	5-methylcytosine
5hmC	5-hydroxymethylcytosine
AF	Allele Frequency
SNPs	Single Nucleotide Polymorphisms
SNVs	Single Nucleotide Variants
IGV	Integrative Genomics Viewer

## References

[1] Stamey T.A., Yang N., Hay A.R., McNeal J.E., Freiha F.S., Redwine E. (1987). Prostate-specific antigen as a serum marker for adenocarcinoma of the prostate. N. Engl. J. Med..

[2] Sundaresan V.M., Smani S., Rajwa P., Renzulli J., Sprenkle P.C., Kim I.Y., Leapman M.S. (2025). Prostate-specific antigen screening for prostate cancer: Diagnostic performance, clinical thresholds, and strategies for refinement. Urol. Oncol..

[3] Canby-Hagino E., Hernandez J., Brand T.C., Troyer D.A., Higgins B., Ankerst D.P., Thompson I.M., Leach R.J., Parekh D.J. (2007). Prostate cancer risk with positive family history, normal prostate examination findings, and PSA less than 4.0 ng/mL. Urology.

[4] Ma C., Ericsson C., Carlsson S.V., Lilja H., Kibel A., Graff R.E., Plym A., Giovannucci E., Mucci L.A., Preston M.A. (2023). Addition of a Genetic Risk Score for Identification of Men with a Low Prostate-specific Antigen Level in Midlife at Risk of Developing Lethal Prostate Cancer. Eur. Urol. Open Sci..

[5] Siegel R.L., Miller K.D., Jemal A. (2018). Cancer statistics, 2018. CA Cancer J. Clin..

[6] Siegel R.L., Miller K.D., Fuchs H.E., Jemal A. (2022). Cancer statistics, 2022. CA Cancer J. Clin..

[7] Raychaudhuri R., Lin D.W., Montgomery R.B. (2025). Prostate Cancer: A Review. JAMA.

[8] Eickelschulte S., Riediger A.L., Angeles A.K., Janke F., Duensing S., Sültmann H., Görtz M. (2022). Biomarkers for the Detection and Risk Stratification of Aggressive Prostate Cancer. Cancers.

[9] Boehm B.E., York M.E., Petrovics G., Kohaar I., Chesnut G.T. (2023). Biomarkers of Aggressive Prostate Cancer at Diagnosis. Int. J. Mol. Sci..

[10] Sekhoacha M., Riet K., Motloung P., Gumenku L., Adegoke A., Mashele S. (2022). Prostate Cancer Review: Genetics, Diagnosis, Treatment Options, and Alternative Approaches. Molecules.

[11] Wilson T.K., Zishiri O.T. (2024). Prostate Cancer: A Review of Genetics, Current Biomarkers and Personalised Treatments. Cancer Rep..

[12] Fontana F., Anselmi M., Limonta P. (2022). Molecular mechanisms and genetic alterations in prostate cancer: From diagnosis to targeted therapy. Cancer Lett..

[13] Vietri M.T., D’Elia G., Caliendo G., Resse M., Casamassimi A., Passariello L., Albanese L., Cioffi M., Molinari A.M. (2021). Hereditary Prostate Cancer: Genes Related, Target Therapy and Prevention. Int. J. Mol. Sci..

[14] Wang A., Shen J., Rodriguez A.A., Saunders E.J., Chen F., Janivara R., Darst B.F., Sheng X., Xu Y., Chou A.J. (2023). Characterizing prostate cancer risk through multi-ancestry genome-wide discovery of 187 novel risk variants. Nat. Genet..

[15] McNevin C.S., Cadoo K., Baird A.-M., Murchan P., Sheils O., McDermott R., Finn S. (2021). Pathogenic BRCA Variants as Biomarkers for Risk in Prostate Cancer. Cancers.

[16] Shah S., Rachmat R., Enyioma S., Ghose A., Revythis A., Boussios S. (2021). BRCA Mutations in Prostate Cancer: Assessment, Implications and Treatment Considerations. Int. J. Mol. Sci..

[17] Khan H.M., Cheng H.H. (2022). Germline genetics of prostate cancer. Prostate.

[18] Marino F., Totaro A., Gandi C., Bientinesi R., Moretto S., Gavi F., Pierconti F., Iacovelli R., Bassi P., Sacco E. (2023). Germline mutations in prostate cancer: A systematic review of the evidence for personalized medicine. Prostate Cancer Prostatic Dis..

[19] Kasuga A., Okamoto T., Udagawa S., Mori C., Mie T., Furukawa T., Yamada Y., Takeda T., Matsuyama M., Sasaki T. (2022). Molecular Features and Clinical Management of Hereditary Pancreatic Cancer Syndromes and Familial Pancreatic Cancer. Int. J. Mol. Sci..

[20] Wang Y., Dai B., Ye D. (2015). CHEK2 mutation and risk of prostate cancer: A systematic review and meta-analysis. Int. J. Clin. Exp. Med..

[21] Kaur H.B., Salles D.C., Murali S., Hicks J., Nguyen M., Pritchard C.C., De Marzo A.M., Lanchbury J.S., Trock B.J., Isaacs W.B. (2020). Genomic and Clinical-Pathologic Characterization of ATM-deficient Prostate Cancer. Clin. Cancer Res..

[22] Beebe-Dimmer J.L., Kapron A.L., Fraser A.M., Smith K.R., Cooney K.A. (2020). Risk of Prostate Cancer Associated with Familial and Hereditary Cancer Syndromes. J. Clin. Oncol..

[23] D’Elia G., Caliendo G., Tzioni M.-M., Albanese L., Passariello L., Molinari A.M., Vietri M.T. (2022). Increased Risk of Hereditary Prostate Cancer in Italian Families with Hereditary Breast and Ovarian Cancer Syndrome Harboring Mutations in BRCA and in Other Susceptibility Genes. Genes.

[24] He W., Xiao Y., Yan S., Zhu Y., Ren S. (2023). Cell-free DNA in the management of prostate cancer: Current status and future prospective. Asian J. Urol..

[25] Urabe F., Sumiyoshi T., Tashiro K., Goto T., Kimura T., Kobayashi T. (2024). Prostate cancer and liquid biopsies: Clinical applications and challenges. Int. J. Urol..

[26] Jahr S., Hentze H., Englisch S., Hardt D., Fackelmayer F.O., Hesch R.D., Knippers R. (2001). DNA fragments in the blood plasma of cancer patients: Quantitations and evidence for their origin from apoptotic and necrotic cells. Cancer Res..

[27] Chen E., Cario C.L., Leong L., Lopez K., Márquez C.P., Chu C., Li P.S., Oropeza E., Tenggara I., Cowan J. (2021). Cell-free DNA concentration and fragment size as a biomarker for prostate cancer. Sci. Rep..

[28] Han D.S.C., Lo Y.M.D. (2021). The Nexus of cfDNA and Nuclease Biology. Trends Genet..

[29] Rahimirad S., Derderian S., Hamel L., Scarlata E., McKercher G., Brimo F., Rajan R., Rompre-Brodeur A., Kassouf W., Sanchez-Salas R. (2025). Refined Procedure to Purify and Sequence Circulating Cell-Free DNA in Prostate Cancer. Int. J. Mol. Sci..

[30] Li W., Liu J.-B., Hou L.-K., Yu F., Zhang J., Wu W., Tang X.-M., Sun F., Lu H.-M., Deng J. (2022). Liquid biopsy in lung cancer: Significance in diagnostics, prediction, and treatment monitoring. Mol. Cancer.

[31] Kopytov S.A., Sagitova G.R., Guschin D.Y., Egorova V.S., Zvyagin A.V., Rzhevskiy A.S. (2025). Circulating Tumor DNA in Prostate Cancer: A Dual Perspective on Early Detection and Advanced Disease Management. Cancers.

[32] Ye Q., Ling S., Zheng S., Xu X. (2019). Liquid biopsy in hepatocellular carcinoma: Circulating tumor cells and circulating tumor DNA. Mol. Cancer.

[33] Parums D.V. (2025). A Review of Circulating Tumor DNA (ctDNA) and the Liquid Biopsy in Cancer Diagnosis, Screening, and Monitoring Treatment Response. Med. Sci. Monit..

[34] Li Q., Huang C.-C., Huang S., Tian Y., Huang J., Bitaraf A., Dong X., Nevalanen M.T., Patel M., Wong J. (2024). 5-hydroxymethylcytosine sequencing in plasma cell-free DNA identifies unique epigenomic features in prostate cancer patients resistant to androgen deprivation therapies. medRxiv.

[35] Sjöström M., Zhao S.G., Levy S., Zhang M., Ning Y., Shrestha R., Lundberg A., Herberts C., Foye A., Aggarwal R. (2022). The 5-Hydroxymethylcytosine Landscape of Prostate Cancer. Cancer Res..

[36] Corradi C., Lencioni G., Gentiluomo M., Felici A., Latiano A., Kiudelis G., van Eijck C.H.J., Marta K., Lawlor R.T., Tavano F. (2023). Polymorphic variants involved in methylation regulation: A strategy to discover risk loci for pancreatic ductal adenocarcinoma. J. Med. Genet..

[37] Toth R., Scherer D., Kelemen L.E., Risch A., Hazra A., Balavarca Y., Issa J.-P.J., Moreno V., Eeles R.A., Ogino S. (2017). Genetic Variants in Epigenetic Pathways and Risks of Multiple Cancers in the GAME-ON Consortium. Cancer Epidemiol. Biomark. Prev..

[38] Ulirsch J., Fan C., Knafl G., Wu M.J., Coleman B., Perou C.M., Swift-Scanlan T. (2013). Vimentin DNA methylation predicts survival in breast cancer. Breast Cancer Res. Treat..

[39] Zeng Y., Jain R., Lam M., Ahmed M., Guo H., Xu W., Zhong Y., Wei G.-H., Xu W., He H.H. (2023). DNA methylation modulated genetic variant effect on gene transcriptional regulation. Genome Biol..

[40] Huang Q., Whitington T., Gao P., Lindberg J.F., Yang Y., Sun J., Väisänen M.-R., Szulkin R., Annala M., Yan J. (2014). A prostate cancer susceptibility allele at 6q22 increases RFX6 expression by modulating HOXB13 chromatin binding. Nat. Genet..

[41] Kechin A., Boyarskikh U., Kel A., Filipenko M. (2017). cutPrimers: A New Tool for Accurate Cutting of Primers from Reads of Targeted Next Generation Sequencing. J. Comput. Biol..

[42] Li H., Durbin R. (2009). Fast and accurate short read alignment with Burrows-Wheeler transform. Bioinformatics.

[43] McKenna A., Hanna M., Banks E., Sivachenko A., Cibulskis K., Kernytsky A., Garimella K., Altshuler D., Gabriel S., Daly M. (2010). The Genome Analysis Toolkit: A MapReduce framework for analyzing next-generation DNA sequencing data. Genome Res..

[44] Van der Auwera G.A., Carneiro M.O., Hartl C., Poplin R., Del Angel G., Levy-Moonshine A., Jordan T., Shakir K., Roazen D., Thibault J. (2013). From FastQ data to high confidence variant calls: The Genome Analysis Toolkit best practices pipeline. Curr. Protoc. Bioinform..

[45] Cibulskis K., Lawrence M.S., Carter S.L., Sivachenko A., Jaffe D., Sougnez C., Gabriel S., Meyerson M., Lander E.S., Getz G. (2013). Sensitive detection of somatic point mutations in impure and heterogeneous cancer samples. Nat. Biotechnol..

[46] Wang K., Li M., Hakonarson H. (2010). ANNOVAR: Functional annotation of genetic variants from high-throughput sequencing data. Nucleic Acids Res..

[47] Pruitt K.D., Brown G.R., Hiatt S.M., Thibaud-Nissen F., Astashyn A., Ermolaeva O., Farrell C.M., Hart J., Landrum M.J., McGarvey K.M. (2014). RefSeq: An update on mammalian reference sequences. Nucleic Acids Res..

[48] Landrum M.J., Lee J.M., Benson M., Brown G.R., Chao C., Chitipiralla S., Gu B., Hart J., Hoffman D., Jang W. (2018). ClinVar: Improving access to variant interpretations and supporting evidence. Nucleic Acids Res..

[49] Gudmundsson S., Singer-Berk M., Watts N.A., Phu W., Goodrich J.K., Solomonson M., Genome Aggregation Database Consortium, Rehm H.L., MacArthur D.G., O’Donnell-Luria A. (2022). Variant interpretation using population databases: Lessons from gnomAD. Hum. Mutat..

[50] Sondka Z., Dhir N.B., Carvalho-Silva D., Jupe S., Madhumita, McLaren K., Starkey M., Ward S., Wilding J., Ahmed M. (2024). COSMIC: A curated database of somatic variants and clinical data for cancer. Nucleic Acids Res..

[51] Liu X., Li C., Mou C., Dong Y., Tu Y. (2020). dbNSFP v4: A comprehensive database of transcript-specific functional predictions and annotations for human nonsynonymous and splice-site SNVs. Genome Med..

[52] Park J., Long D.T., Lee K.Y., Abbas T., Shibata E., Negishi M., Luo Y., Schimenti J.C., Gambus A., Walter J.C. (2013). The MCM8-MCM9 complex promotes RAD51 recruitment at DNA damage sites to facilitate homologous recombination. Mol. Cell. Biol..

[53] Gao X.-P., Dong J.-J., Xie T., Guan X. (2021). Integrative Analysis of MUC4 to Prognosis and Immune Infiltration in Pan-Cancer: Friend or Foe?. Front. Cell Dev. Biol..

[54] Singh A.P., Chauhan S.C., Bafna S., Johansson S.L., Smith L.M., Moniaux N., Lin M.-F., Batra S.K. (2006). Aberrant expression of transmembrane mucins, MUC1 and MUC4, in human prostate carcinomas. Prostate.

[55] Hustedt N., Saito Y., Zimmermann M., Álvarez-Quilón A., Setiaputra D., Adam S., McEwan A., Yuan J.Y., Olivieri M., Zhao Y. (2019). Control of homologous recombination by the HROB-MCM8-MCM9 pathway. Genes Dev..

[56] Helderman N.C., Terlouw D., Bonjoch L., Golubicki M., Antelo M., Morreau H., van Wezel T., Castellví-Bel S., Goldberg Y., Nielsen M. (2023). Molecular functions of MCM8 and MCM9 and their associated pathologies. iScience.

[57] Zhang W., van Gent D.C., Incrocci L., van Weerden W.M., Nonnekens J. (2020). Role of the DNA damage response in prostate cancer formation, progression and treatment. Prostate Cancer Prostatic Dis..

[58] Schiewer M.J., Knudsen K.E. (2019). DNA Damage Response in Prostate Cancer. Cold Spring Harb. Perspect. Med..

[59] Morii I., Iwabuchi Y., Mori S., Suekuni M., Natsume T., Yoshida K., Sugimoto N., Kanemaki M.T., Fujita M. (2019). Inhibiting the MCM8-9 complex selectively sensitizes cancer cells to cisplatin and olaparib. Cancer Sci..

[60] Wood-Trageser M.A., Gurbuz F., Yatsenko S.A., Jeffries E.P., Kotan L.D., Surti U., Ketterer D.M., Matic J., Chipkin J., Jiang H. (2014). MCM9 mutations are associated with ovarian failure, short stature, and chromosomal instability. Am. J. Hum. Genet..

[61] Bhatia R., Siddiqui J.A., Ganguly K., Thompson C.M., Cannon A., Aithal A., Perumal N., Maurya S.K., Li X., Cox J.L. (2023). Muc4 loss mitigates epidermal growth factor receptor activity essential for PDAC tumorigenesis. Oncogene.

[62] King R.J., Yu F., Singh P.K. (2017). Genomic alterations in mucins across cancers. Oncotarget.

[63] Mateo J., Carreira S., Sandhu S., Miranda S., Mossop H., Perez-Lopez R., Nava Rodrigues D., Robinson D., Omlin A., Tunariu N. (2015). DNA-Repair Defects and Olaparib in Metastatic Prostate Cancer. N. Engl. J. Med..

[64] Matsumoto T., Shiota M., Blas L., Eto M. (2022). Role of Olaparib in the Management of Metastatic Castration-Resistant Prostate Cancer: A Japanese Clinician’s Perspective. Cancer Manag. Res..

[65] Taylor A.K., Kosoff D., Emamekhoo H., Lang J.M., Kyriakopoulos C.E. (2023). PARP inhibitors in metastatic prostate cancer. Front. Oncol..

[66] Liu K., Wang Y., Zhu Q., Li P., Chen J., Tang Z., Shen Y., Cheng X., Lu L.-Y., Liu Y. (2020). Aberrantly expressed HORMAD1 disrupts nuclear localization of MCM8-MCM9 complex and compromises DNA mismatch repair in cancer cells. Cell Death Dis..

[67] Belhadj S., Terradas M., Munoz-Torres P.M., Aiza G., Navarro M., Capellá G., Valle L. (2020). Candidate genes for hereditary colorectal cancer: Mutational screening and systematic review. Hum. Mutat..

[68] Ahmed M., Soares F., Xia J.-H., Yang Y., Li J., Guo H., Su P., Tian Y., Lee H.J., Wang M. (2021). CRISPRi screens reveal a DNA methylation-mediated 3D genome dependent causal mechanism in prostate cancer. Nat. Commun..

[69] Chen Y.-T., Panarelli N.C., Piotti K.C., Yantiss R.K. (2014). Cancer-testis antigen expression in digestive tract carcinomas: Frequent expression in esophageal squamous cell carcinoma and its precursor lesions. Cancer Immunol. Res..

[70] Ding Y., Wang H., Cao W., Cao T., Jiang H., Yu Z., Zhou Y., Xu M. (2024). TTC22 as a potential prognostic marker and therapeutic target in pancreatic cancer: Insights into immune infiltration and epithelial-mesenchymal transition. Oncol. Lett..

[71] You A., Tian W., Yuan H., Gu L., Zhou J., Deng D. (2022). TTC22 promotes m6A-mediated WTAP expression and colon cancer metastasis in an RPL4 binding-dependent pattern. Oncogene.

[72] Baryshev M., Vjaters E. (2025). Allele-Specific CG/CCWGG Methylation of the PSA Promoter Discriminates Aggressive, Indolent, and Benign Prostate Cell Lines and Is Involved in the Regulation of PSA Expression. Int. J. Mol. Sci..

[73] Feng D., Zhu W., Shi X., Xiong Q., Li D., Wei W., Han P., Wei Q., Yang L. (2022). Spindle and kinetochore-associated complex subunit 3 could serve as a prognostic biomarker for prostate cancer. Exp. Hematol. Oncol..

[74] Vlasschaert C., Mack T., Heimlich J.B., Niroula A., Uddin M.M., Weinstock J., Sharber B., Silver A.J., Xu Y., Savona M. (2023). A practical approach to curate clonal hematopoiesis of indeterminate potential in human genetic data sets. Blood.

[75] Dong D., Wang Z., Liu M., Zhang Q., Xu W., Wei Y., Zhu J., Yang X., Zhang Q., Zhu Y. (2025). Combined SNPs sequencing and allele specific proteomics capture reveal functional causality underpinning the 2p25 prostate cancer susceptibility locus. Nat. Commun..

[76] Zhang P., Xia J.-H., Zhu J., Gao P., Tian Y.-J., Du M., Guo Y.-C., Suleman S., Zhang Q., Kohli M. (2018). High-throughput screening of prostate cancer risk loci by single nucleotide polymorphisms sequencing. Nat. Commun..

[77] Ci X., Chen S., Zhu R., Zarif M., Jain R., Guo W., Ramotar M., Gong L., Xu W., Singh O. (2024). Oral pimonidazole unveils clinicopathologic and epigenetic features of hypoxic tumour aggressiveness in localized prostate cancer. BMC Cancer.

